# Impact of COVID-19 control on lung, breast, and colorectal pathological cancer diagnoses. A comparison between the Netherlands, Aotearoa New Zealand, and Northern Ireland

**DOI:** 10.1186/s12885-023-11216-3

**Published:** 2023-07-26

**Authors:** Helen Mitchell, Jennifer Mclean, Anna T Gavin, Otto Visser, Elinor Millar, Tessa Luff, Damien Bennett

**Affiliations:** 1 Centre for Public Health, Northern Ireland Cancer Registry, Queen’s University Belfast, Belfast, Northern Ireland; 2 Centre for Public Health, Queen’s University Belfast, Belfast, Northern Ireland; 3 Department of Registration, Netherlands Comprehensive Cancer Organisation, Utrecht, Netherlands; 4 Te Aho o Te Kahu – Cancer Control Agency, Wellington, New Zealand

**Keywords:** COVID-19, Cancer, Population-based, Pathology, Registry

## Abstract

**Background:**

The COVID-19 pandemic was managed in Aotearoa New Zealand (NZ) by a COVID-19 elimination policy, involving border closure and an initial national lockdown. This was different to most other countries including Northern Ireland (NI) and the Netherlands (NED). We quantify the effect of these policies on the diagnosis of three major cancers, comparing NZ with these two European countries.

**Method:**

Data from NED, NZ and NI population-based cancer registries were used to assess trends in all pathologically diagnosed (PD) lung, breast, and colorectal cancers from March to December 2020 (pandemic period) and compared to the similar pre-pandemic period (2017–2019). Trend data were also collated on COVID-19 cases and deaths per 100,000 in each population.

**Results:**

Comparing the pre-pandemic period to the pandemic period there were statistically significant reductions in numbers of lung (↓23%) and colorectal (↓15%) PD cancers in NI and numbers of breast (↓18%) and colorectal cancer (↓18.5%) diagnosed in the NED. In NZ there was no significant change in the number of lung (↑10%) or breast cancers (↑0.2%) but a statistically significant increase in numbers of colorectal cancer diagnosed (↑5%).

**Conclusion:**

The impact of COVID-19 on cancer services was mitigated in NZ as services continued as usual reflecting minimal healthcare disruption and protected cancer services linked with the elimination approach adopted. The reduction in PD cases diagnosed in NED and NI were linked with higher COVID-19 rates and reflect societal restrictions which resulted in delayed patient presentation to primary and secondary care, disruption to screening and healthcare services as a result of COVID-19 infections on staff and the need to shift intensive care to COVID-19 patients. Reductions in PD cancers in NI and the NED and in particularly lung cancers in NI, highlight the need for targeted public health campaigns to identify and treat ‘missing’ patients. Protecting cancer services should be a priority in any future pandemic or systemic healthcare system disruption.

**Supplementary Information:**

The online version contains supplementary material available at 10.1186/s12885-023-11216-3.

## Background

The World Health Organisation (WHO) declared the novel coronavirus COVID-19 pandemic on 11th March 2020 [1]. The pandemic has had a substantial and ongoing impact on all aspects of society including education, commerce and healthcare systems [2]. As of 28th February 2023 over 758 million cases and over 6.8 million deaths had been reported worldwide [3]. The approach to the pandemic differed between the countries studied with NZ adopting a COVID-19 elimination policy, which involved border closure and an initial national lockdown, [4] while NI and NED adopted a containment and mitigation policy involving only limited travel and quarantine measures [5].

Cancer patients have been adversely impacted during the COVID-19 pandemic due to delays and rescheduling of cancer treatments and appointments [6]. In addition to the impact on current cancer patients, non-critical care such as cancer screening services were also disrupted. Although cessation of cancer screening was for a relatively short period, on reopening these services had to manage the backlog created and most were unable to operate at pre-pandemic capacity due to enhanced infection prevention and control and cleaning procedures, staff absence and redeployment associated with high levels of COVID-19 community transmission [7]. The impact of COVID-19 on screening varied across countries in this study. In NI breast, colorectal and cervical screening services were paused for a period of four months [8], in NED, faecal immunochemical testing (FIT) for colorectal cancer was paused for a period of 11 weeks [7] while breast screening was paused for a period of 12 weeks [9]. In NZ bowel, breast and cervical screening programmes were paused for 4 weeks in March and April 2020 [4]. Apart from temporary cessation of their screening programmes, disruption to health care services in NZ were minimal due to NZ’s strict COVID-19 elimination strategy,

The purpose of this study is to investigate the impact of COVID-19 on three common cancers (lung, breast, colorectal) in three high-income countries (NZ, NED and NI), which had different approaches to COVID-19 containment using population level data between the pre-pandemic period (2017–2019 average) and the initial pandemic period (2020).

## Research in context

### Evidence before this study

A previous study found NI experienced a 23% reduction in the number of pathologically diagnosed cancers between 1st March and 12th September 2020 compared to the previous three years (2017–2019) [10]. Although Hamilton et al. reported some recovery between August and September, the number of PD lung cancers remained well below pre-COVID-19 levels. An observational study from the NED Cancer Registry (NCR) also found fewer PD cancers (average weekly reduction 20%, range 9 − 27%) in the initial seven weeks of the pandemic (weeks 9 to 15, Feb 24th to April 12th) compared to the preceding seven weeks (weeks 2 to 8) [11]. In NZ there was a decline in cancer registrations during the initial national lockdown period (March-April 2020); however registrations returned to pre-shutdown levels over the subsequent months [4]. Previous studies investigated the impact of COVID-19 on colorectal and breast cancers. Gathani et al. [12] found that during the first six months of 2020 in England, there was a 28% reduction in breast cancer referrals and a 16% decrease in patients receiving their first treatment, compared to the same period in 2019. A study by Filipe et al. [13] found that pausing of the breast screening programme in NED led to a reduction in breast surgery for earlier stage cancers. A population-based study in England reported 3500 fewer colorectal cancer patients diagnosed in April-October 2020 compared to the same period in 2019 and found a 31% relative reduction in surgery in April 2020 [14]. Thirty six hospitals across the UK and Ireland reported deviation from national guidelines for colorectal cancer at all points of the patient care pathway during the first part of the COVID-19 pandemic [15].

### Added value of this study

This study identifies, using population-based data, the impact of the first period of the COVID-19 pandemic on healthcare services. It compares for the first time the impact of different approaches to pandemic management on cancer services and identifies the scale of ‘lost cases’.

The impact on breast and colorectal cancers includes that from alterations to screening services, while for lung cancers the overlap of respiratory symptoms with COVID-19 posed problems for case identification, investigation, and treatment.

## Methods

National cancer registries in each country provided population level data on pathologically diagnosed cancers for the pre-pandemic period (2017–2019) and the pandemic period (2020). Information was also collated on the number of COVID-19 cases and deaths per 100,000 population in each country – in the NED from the government coronavirus dashboard [16], in NZ from the Ministry of Health’s COVID-19 Dashboard [17], and in NI from the Department of Health Website [18].

The International Classification of Diseases, 10th revision (ICD10) codes used to identify cancers in NI were as follows; lung (C33-C34) female breast (C50) and colorectal (C18-C20). These data were derived from pathology data provided monthly to NICR [19]. For the NED the Cancer Registry (NCR) was also the source of pathologically diagnosed cancers and the ICD10 codes used were as follows; breast including male and female cancer (C50) and Ductal Carcinoma in situ (DCIS) (D05), lung including thymus and pleura (C33, C34, C37 and C38.4) and colorectal (C18-C20). The NZ data was from the NZ Cancer Registry (NZCR), with colorectal classification (C18-C20) similar to NED and NI, breast including male and female cases (C50) and lung (C30 – C39) including nasal cavity, (although the majority (94.2%) were classified as lung cancer (C33-C34)).

COVID-19 cases and deaths per 100,000 were calculated using midterm population estimates. Comparisons were made between average monthly pathology samples for the pre-pandemic period (2017–2019) and the pandemic period (2020) with differences expressed as percentages.

### Inclusion and exclusion criteria

The same inclusion and exclusion criteria were applied to both the pre-pandemic and pandemic periods. In NI, pathology samples processed by private laboratories were excluded, this makes up a very small number of samples processed in NI. Where a patient has more than one pathology sample of the same cancer type this was counted once, except in cases where the pathology samples are separated by a period greater than two years. In NED, where a patient has more than one pathology sample of the same cancer type this was counted once. Only the first diagnosis per patient is included for incidence of invasive breast cancer, but *all* diagnoses for DCIS are included. One laboratory was excluded from data provided by NED due to delayed pathology notifications. In NZ, where a patient has more than one pathology sample of the same cancer type this was counted once.

### Statistical analysis

Data analysis was completed using SPSS (IBM SPSS Statistics V25). Mann-Whitney U tests were performed to compare the number of cancer cases in the pre-pandemic period (2017–2019) and the pandemic period (2020) (two independent samples). Correlations were also calculated between monthly COVID-19 deaths and monthly cancer cases in each country.

## Results

**Fig. 1:**
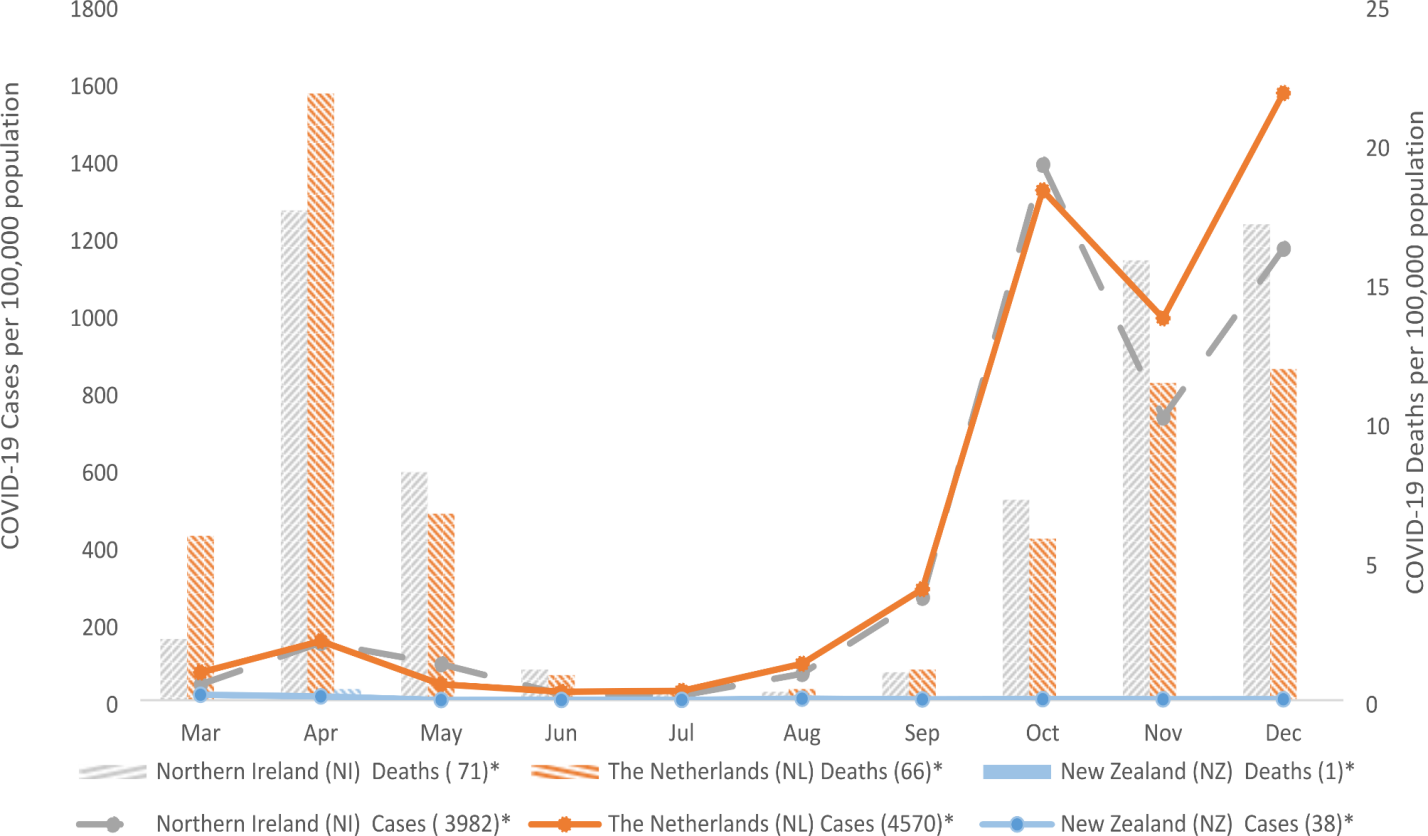
COVID-19 cases and deaths, per 100k population, 2020^*Totals per 100,000 population^

In 2020 highest monthly COVID-19 cases in NI was 1386/100K in October, in the NED it was 1571/100K in December, while in NZ it was 14/100K in March. Lower COVID-19 cases were recorded in mid-2020 following severe initial lockdown measures in all countries, with national minimum case numbers of 12/100K in NI in July, 22/100K in the NED in June and 0.3/100K in NZ in May. The highest number of deaths per month were reported in April in each country (NI (16.8), the NED (21.9) and NZ (0.4)) and the lowest number of deaths were reported in July and August in NI (both 0.3) and in July in the NED (0.3), with NZ recording no deaths for most months (Fig. 1).

### Breast cancers

**Fig. 2:**
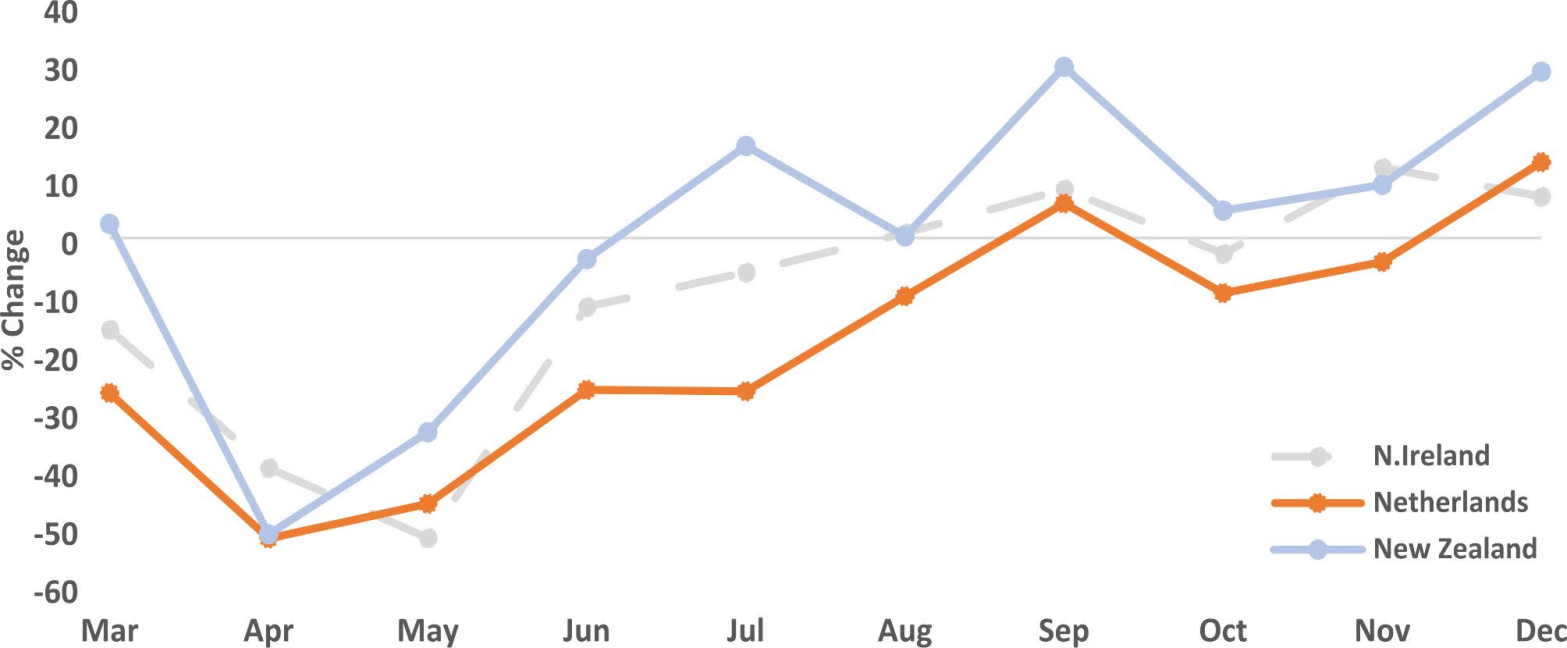
Difference between pathologically diagnosed breast cancer during the pre-pandemic (2017–2019 Avg) and pandemic (2020) periods by month and country

In 2020 there was a 10% reduction overall in PD breast cancer diagnoses in NI compared to the pre-pandemic period with greatest reductions in April (40%) and May (52%), with broad recovery from August onward. NED reported a similar pattern to NI with an overall reduction in pathologically diagnosed breast cancers of 18.2%, greatest reductions in April (52%) and May (46%) and recovery by September 2020. In contrast, NZ recorded a very small (0.2%) increase in PD breast cancers in 2020 compared to the pre-pandemic period with initial large decreases in April (51%) and May (34%) followed by early recovery by June 2020 with PDs consistently at or above pre-pandemic levels (Fig. 2).

### Lung cancer

**Fig. 3:**
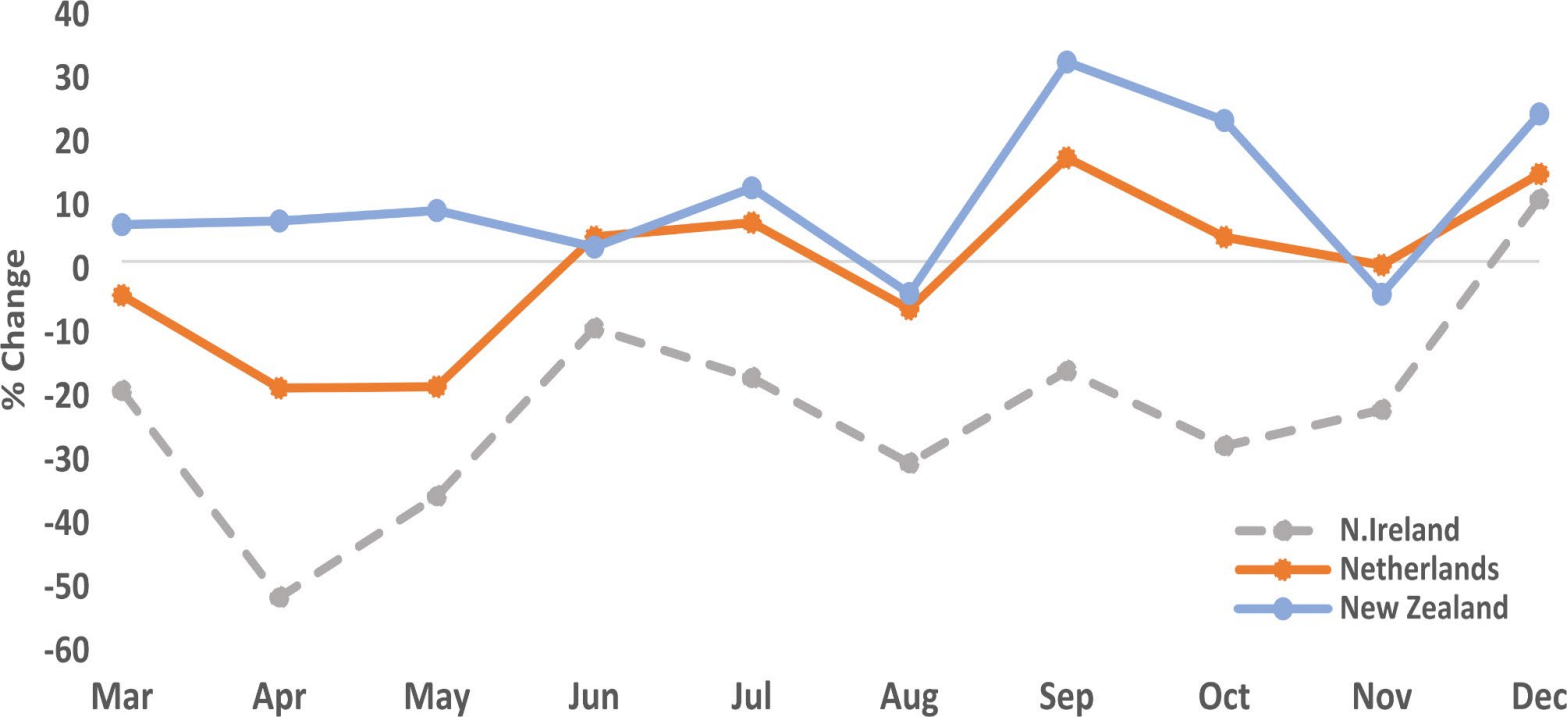
Difference between pathological diagnosed lung cancers during the pre-pandemic (2017–2019 Avg) and pandemic (2020) periods by month and country

Although long-term global trends predicted lung cancer incidence would increase in 2020 [20] with the onset of the COVID-19 pandemic reductions in PD lung cancers were recorded in some countries. NI experienced an overall reduction of 23% during 2020 with the greatest reduction in April (53%) and only partial recovery for the rest of the year. Between March and December 2020, the reduction in number of lung cancer diagnoses was statistically significant (p = 0.001) in NI compared to the pre-COVID period. The NED experienced a very small overall reduction in pathologically diagnosed lung cancers (1%) with the greatest reductions in April and May (both 20%) but broad recovery by June 2020. In contrast, NZ recorded a 10% overall increase in lung cancers in 2020 compared to the pre-pandemic period, with only very slight reductions recorded in August and November (Fig. 3) and an increase recorded in the period between March and June 2020.

### Colorectal (CR) cancers

**Fig. 4:**
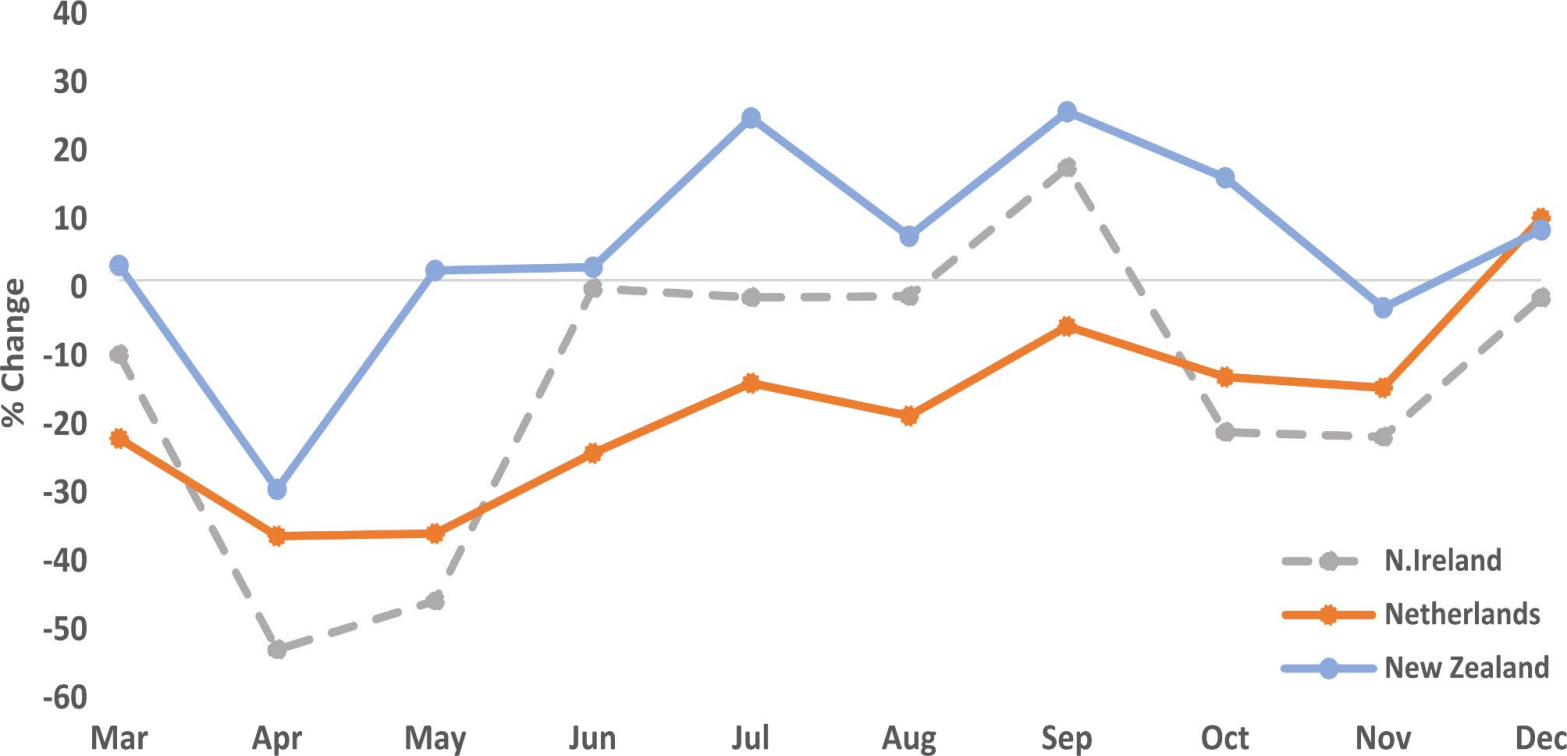
Difference between pathologically diagnosed colorectal cancers during the pre-pandemic (2017–2019 Avg) and pandemic (2020) periods by month and country

The overall reduction in PD CR cancers during 2020 in NI was 15% with the greatest reductions in April (54%) and May (47%) (Fig. 3). There was good recovery between June and September, with PD cancers returning to at least pre-pandemic levels, although this was followed by reductions again in October and November. The NED recorded an overall reduction of 18.5% in 2020, with the greatest reductions in April and May (both 37%) and only partial recovery by July, with levels remaining about 20% lower than pre-pandemic levels up to Nov 2020. Again, in contrast, NZ recorded a 5% overall increase in PD CR cancers in 2020 compared to the pre-pandemic period, with a 31% reduction in April followed by an immediate recovery by May with this rebound sustained for the rest of the year and PD were generally above pre-pandemic levels (Fig. 4).

## Discussion

Colorectal, breast and lung cancer are three of the most common cancers (excluding Non-Melanoma Skin Cancers) and this study reports the impact of different national pandemic control mechanisms on levels of these PD cancers. Prior to the pandemic, cancer trends predicted increases in new cancer rates, due in part to the ageing population and population growth, except where screening has been shown to decrease colorectal cancer levels [2]. Therefore, the lower levels found in this study were unexpected and can be directly linked to the COVID-19 pandemic.

Although long term global trends predicted lung cancer incidence would increase in 2020 [20], only NZ recorded an increase in PD lung cancers. NZ also recorded slight increases in breast and colorectal cancer despite the initial reductions at the start of the pandemic.

In NZ the FIT bowel screening programme began in July 2017, with the last regions joining in June 2022 [21]. The increase in colorectal cancer registrations in NZ may be linked to the introduction of bowel screening. Increases in cancer registrations were not found for any cancers in either the NED or NI.

The response to COVID-19 imposed sudden major change on health services as they rapidly adapted to meet the demands of the novel coronavirus. Primary care interactions switched from in-person to virtual consultations with multiple levels of telephone triage [22, 23]. Non-essential services such as cancer screening were paused and staff redeployed to care for COVID-19 patients, who had longer stays in high dependency and intensive care facilities compared to other respiratory conditions such as influenza [24]. In NI and NED, COVID-19 responses included legislation to restrict population mixing, ‘stay at home messaging’ and school and non-essential workplace closures, while in NZ the elimination approach entailed more severe restrictions including border closure and mandatory quarantine.

The impact of COVID-19 on breast, colorectal and lung cancers differed. Breast and colorectal cancers have population-based screening programmes in all countries included in this study. Screening accounts for approximately 30% of new breast cancers and 8% of colorectal cancers diagnosed in NI [25]. It is likely that pauses in cancer screening contributed to reduction in patient presentations during 2020, however this impact varies across the countries studied and there would not have had a uniform effect due to the differences in the screening ages and testing used. In NED, FIT has been fully implemented in the population since 2019 for those aged 55–75 years old [7]. Until 2021, NI used the faecal occult blood test (FOBT) for adults aged 60 to 74 and has since moved to FIT which is potentially more accurate, in part due to better patient acceptability and fewer false negatives [26].

Lung cancer was particularly impacted in NI with an overall reduction of 23% compared to just 1% in NED. While there is currently no population-based screening for lung cancer, some countries have piloted lung cancer screening for those most at risk of developing lung cancer. In NED, the NELSON study, a Dutch-Belgian Randomized Lung Cancer Screening Trial was conducted between 2004 and 2015 to investigate screening for high-risk subjects and found that there was a decrease in 10-year lung cancer mortality of at least 25% compared to the control group. However there was no statistically significant difference in all-cause mortality at 10 years between the screened and unscreened groups [27]. NZ does not have any lung cancer screening at present and there were no pilots during the study period (Personal communication, 2023). In the UK, there have been pilot lung cancer screening programs, however these are less developed in NI compared to England (UK Lung Cancer Coalition (UKLCC), 2020). During the initial first wave of the pandemic, there were significant pressures on respiratory teams to lead the diagnosis and treatment of COVID-19 patients which may have reduced their capacity to manage possible lung cancer diagnoses [28]. There were also concerns that Computerised Tomography (CT) imaging findings of COVID-19 may be radiologically indistinguishable from patients with lung cancer who have developed pneumonia [29].

The respiratory symptoms of lung cancer and COVID–19, including new persistent cough and breathlessness overlap. There were concerns regarding conflicting public health messaging from governments, with the UK government urging people to “Stay at Home” whilst simultaneously promoting better lung cancer awareness [28]. Across the UK the UKLCC reported a 75% reduction in urgent lung cancer referrals during the first wave of the pandemic [30], suggesting patients may have decided to self-isolate instead of seeking medical care when respiratory symptoms began. As a result of the reduction in urgent referrals for suspected lung cancer cases and pathology samples, some have called for lung cancer screening to be included in follow-up appointments for COVID-19 patients who have residual respiratory symptoms after the infection has cleared [31]. Lung cancer patients are also at increased risk of COVID-19, which may have led to missed or delayed diagnoses of lung cancers when a COVID-19 diagnosis may have been suspected, especially with high COVID-19 prevalence at the early stages of the pandemic [32]. In stark contrast, NZ had a 10% increase in the number of PD lung cancers detected in 2020 which occurred against a background of very low levels of COVID-19 and high levels of testing. There was a considerable dedication of resources to ensure the continuation of cancer services in NZ, with monitoring and evaluation of service delivery at the local level. Particular attention was placed on ensuring no further exacerbation of existing health inequalities for Māori and Pacific peoples [33]. This suggests that in NZ patients were more likely to attend healthcare settings as the likelihood of COVID-19 infection was low and also that lung cancer symptoms such as a persistent cough and breathlessness were perhaps investigated more thoroughly.

In NI, it is estimated that there were about 77 ‘missing’ lung cancer patients when cancer registrations were complete [34], which are likely to lead to later disease stage at presentation and may translate to reductions in survival. This ‘missing’ figure takes into account the other methods of diagnosis and not just PD cancers. In addition, a recent study conducted by Monari et al. [35] found that patients with cancer were at increased risk of hospitalisation and/or death from COVID-19. There may therefore be patients who had an undiagnosed cancer who went on to die from COVID-19 due to their increased vulnerability. Cancer Registries will be key to monitoring the impact of COVID-19 for many years.

## Conclusions

COVID-19 had a lasting impact on detection and treatment of cancer patients, the full extent of which has yet to be quantified. This study has identified a concerning reduction in the number of lung cancers identified pathologically in NI compared to both NED and NZ. This is likely to be due to reductions in patient interaction with primary and hospital care and the overlap of lung cancer symptoms with COVID-19, possibly resulting in people not seeking medical attention.

It has been reported that countries which sought to eliminate COVID-19, like NZ, registered fewer deaths, had fewer restrictions and lockdowns and likely had much less disruption of healthcare and cancer services [5]. Our study found that in NZ, a country which sought to eliminate COVID-19 in the early stages of the pandemic and had very low rates of COVID-19 transmission during 2020, experienced very little change in levels of pathologically diagnosed breast, colorectal and lung cancers. In contrast two European countries (NI and NED) which adopted COVID-19 policies focused on containment and mitigation, rather than elimination, had high rates of COVID-19 transmission and experienced substantial reductions in pathologically diagnosed cancers. NI experienced reductions of 10%, 15% and 23% in breast, colorectal and lung cancers respectively while the NED experienced reductions of 18%, 19% and 1% in these same cancers. Longer term follow-up studies are required to assess the extent of recovery in cancer diagnoses across these countries.

## Strengths

The strengths of this study include the use of national population-based cancer registry data and comparison with an average of three previous years to reduce inter-year variability.

## Limitations

As pathological data was used, these will not be as complete or rigorously validated as finalised cancer registration data. However, data for 2017–2019 was also based on pathological diagnoses of cancer to ensure consistency of comparator data. As bowel screening roll-out was not uniform across NZ during the period of study, this may present a challenge in interpreting changes in colorectal cancer registrations during this time.

Also, the proportion of cancers that have a pathological diagnosis (microscopically verified) varies by cancer type. For example, in 2018 in the UK, 99% of breast cancers, 89% of colorectal cancers and 70% of lung cancers were microscopically verified (UKIACR, 2021). In NED 99.8% of breast cancers, 96% of colorectal cancers and 82% of lung cancers were microscopically verified.

The ICD10 codes used to define each cancer site varied slightly between the three countries investigated. For example, the NED included in-situ breast (D05) within their breast cancer cases while NZ included thymus, pleura, and nasal cavity (C30 – C39) in their lung cancer case numbers. Therefore, direct comparisons of cancer case numbers between countries should be interpreted with caution and percentage changes within each country over the period of investigation has been used throughout.

## Electronic supplementary material

Below is the link to the electronic supplementary material.

Supplementary Material 1

## Data Availability

The datasets generated and/or analysed during the current study are not publicly available due the limits of the ethical approval granted to the NICR, NCR and NZCR to share patient level data. Anonymised, non-patient level data can be made available from the corresponding author on reasonable request.

## Abbreviations

CR: Colorectal cancer
CT: Computerised tomography
DCIS: Ductal carcinoma in situ
FIT: Faecal immunochemical test
FOBT: Faecal occult blood test
ICD10: International Classification of Diseases, 10th revision
NCR: Netherlands Cancer Registry
NED: Netherlands
NI: Northern Ireland
NICR: Northern Ireland Cancer Registry
NZ: Aotearoa New Zealand
NZCR: Aotearoa New Zealand Cancer Registry
PD: Pathologically diagnosed
UKLCC: UK Lung Cancer Coalition
