# Supplementary material for: Impact of COVID-19 control on lung, breast, and colorectal pathological cancer diagnoses. A comparison between the Netherlands, Aotearoa New Zealand, and Northern Ireland
Source: BMC Cancer. 2023 Jul 26;23:700. doi: 10.1186/s12885-023-11216-3 (PMC10373228; doi:10.1186/s12885-023-11216-3)
Supplement: Supplementary file 1 — Supplementary Material 1 [file 12885_2023_11216_MOESM1_ESM.docx]

**Supplementary Material**

**Supplementary Material, Table 1**: *COVID cases and deaths per 100,000 persons in each country in 2020.*

| **Month** | **Northern Ireland (NI)** | | **The Netherlands (NED)** | | **New Zealand (NZ)** | |
| --- | --- | --- | --- | --- | --- | --- |
|  | **Cases** | **Deaths** | **Cases** | **Deaths** | **Cases** | **Deaths** |
| **Jan** | 0.0 | 0.0 | 0.0 | 0.0 | 0.0 | 0.0 |
| **Feb** | 0.0 | 0.0 | 0.0 | 0.0 | 0.02 | 0.0 |
| **Mar** | 40.7 | 2.2 | 71.7 | 5.9 | 13.9 | 0.0 |
| **Apr** | 148.7 | 17.6 | 152.7 | 21.8 | 9.7 | 0.4 |
| **May** | 93.2 | 8.2 | 40.9 | 6.7 | 0.3 | 0.1 |
| **Jun** | 14.9 | 1.1 | 21.7 | 0.9 | 0.5 | 0.0 |
| **Jul** | 12.2 | 0.3 | 24.0 | 0.3 | 0.7 | 0.0 |
| **Aug** | 68.2 | 0.3 | 94.1 | 0.4 | 3.8 | 0.0 |
| **Sep** | 265.2 | 1.0 | 287.3 | 1.1 | 1.9 | 0.1 |
| **Oct** | 1385.7 | 7.2 | 1319.0 | 5.8 | 2.5 | 0.0 |
| **Nov** | 731.5 | 15.8 | 988.1 | 11.4 | 2.1 | 0.0 |
| **Dec** | 1168.1 | 17.1 | 1570.6 | 11.9 | 2.2 | 0.0 |

| **Colorectal Cancer** | | | |
| --- | --- | --- | --- |
| **Month** | **Northern Ireland (NI)** | **The Netherlands (NED)** | **New Zealand (NZ)** |
| **Jan** | +14.94 | -13.40 | +31.43 |
| **Feb** | +7.59 | -15.83 | +21.10 |
| **Mar** | -10.87 | -23.14 | +2.16 |
| **Apr** | -54.02 | -37.42 | -30.58 |
| **May** | -46.88 | -37.04 | +1.42 |
| **Jun** | -1.19 | -25.29 | +1.90 |
| **Jul** | -2.53 | -15.06 | +23.72 |
| **Aug** | -2.35 | -19.85 | +6.43 |
| **Sep** | +16.47 | -6.75 | +24.63 |
| **Oct** | -22.22 | -14.15 | +14.98 |
| **Nov** | -22.92 | -15.74 | -4.03 |
| **Dec** | -2.56 | +9.07 | +7.36 |
|  |  |  |  |
| Overall % change Mar – Dec | -14.91 | -18.54 | +4.80 |

**Supplementary Material, Table 2:** *Percentage (%) change in Colorectal cancer pathological diagnoses between an average of 2017-2019 and* 2020

| **Breast Cancer** | | | |
| --- | --- | --- | --- |
| **Month** | **Northern Ireland (NI)** | **The Netherlands (NED)** | **New Zealand (NZ)** |
| Jan | -12.69 | +6.66 | +11.19 |
| Feb | +18.75 | +5.07 | +10.80 |
| Mar | -15.79 | -26.72 | +2.43 |
| Apr | -39.66 | -51.80 | -51.08 |
| May | -51.72 | -45.83 | -33.50 |
| Jun | -11.86 | -26.18 | -3.63 |
| Jul | -5.93 | -26.44 | +15.86 |
| Aug | +0.83 | -10.05 | +0.26 |
| Sep | +8.40 | +5.95 | +29.50 |
| Oct | -2.72 | -9.52 | +4.71 |
| Nov | +12.10 | -4.14 | +9.16 |
| Dec | +7.14 | +13.04 | +28.66 |
|  |  |  |  |
| Overall % change Mar – Dec | -9.92 | -18.17 | +0.24 |

**Supplementary Material, Table 3:** *Percentage (%) change in Breast cancer pathological diagnoses between an average of 2017-2019 and* 2020

| **Lung Cancer** | | | |
| --- | --- | --- | --- |
| **Month** | **Northern Ireland (NI)** | **The Netherlands (NED)** | **New Zealand (NZ)** |
| **Jan** | +6.78 | +2.14 | +25.98 |
| **Feb** | +28.00 | +2.94 | +0.68 |
| **Mar** | -20.37 | -5.33 | +5.78 |
| **Apr** | -52.83 | -19.92 | +6.35 |
| **May** | -36.92 | -19.71 | +8.00 |
| **Jun** | -10.53 | +3.86 | +2.24 |
| **Jul** | -18.33 | +6.10 | +11.56 |
| **Aug** | -31.75 | -7.50 | -5.06 |
| **Sep** | -17.24 | +16.29 | +31.34 |
| **Oct** | -29.03 | +3.81 | +22.15 |
| **Nov** | -23.33 | -0.59 | -5.16 |
| **Dec** | +9.76 | +13.74 | +23.18 |
|  |  |  |  |
| Overall % change Mar – Dec | -23.06 | -0.93 | +10.04 |

**Supplementary Material, Table 4:** *Percentage (%) change in Lung cancer pathological diagnoses between an average of 2017-2019 and* 2020

**Supplementary Material, Figure 1:** *Percentage (%) change in diagnoses for breast, lung and colorectal cancer, alongside line graph showing number of COVID-19 deaths per 100k persons in* ***Northern Ireland***

**Supplementary Material, Figure 2:** *Percentage (%) change in diagnoses for breast, lung and colorectal cancer, alongside line graph showing number of COVID-19 deaths per 100k persons in* ***The Netherlands***

**Supplementary Material, Figure 3:** *Percentage (%) change in diagnoses for breast, lung and colorectal cancer, alongside line graph showing number of COVID-19 deaths per 100k persons in* ***New Zealand***
